# Circuit Mechanisms Governing Local vs. Global Motion Processing in Mouse Visual Cortex

**DOI:** 10.3389/fncir.2017.00109

**Published:** 2017-12-22

**Authors:** Rune Rasmussen, Keisuke Yonehara

**Affiliations:** The Danish Research Institute of Translational Neuroscience—DANDRITE, Nordic EMBL Partnership for Molecular Medicine, Department of Biomedicine, Aarhus University, Aarhus, Denmark

**Keywords:** visual cortex, direction selectivity, local motion, global motion, pattern cell, component cell

## Abstract

A withstanding question in neuroscience is how neural circuits encode representations and perceptions of the external world. A particularly well-defined visual computation is the representation of global object motion by pattern direction-selective (PDS) cells from convergence of motion of local components represented by component direction-selective (CDS) cells. However, how PDS and CDS cells develop their distinct response properties is still unresolved. The visual cortex of the mouse is an attractive model for experimentally solving this issue due to the large molecular and genetic toolbox available. Although mouse visual cortex lacks the highly ordered orientation columns of primates, it is organized in functional sub-networks and contains striate- and extrastriate areas like its primate counterparts. In this Perspective article, we provide an overview of the experimental and theoretical literature on global motion processing based on works in primates and mice. Lastly, we propose what types of experiments could illuminate what circuit mechanisms are governing cortical global visual motion processing. We propose that PDS cells in mouse visual cortex appear as the perfect arena for delineating and solving how individual sensory features extracted by neural circuits in peripheral brain areas are integrated to build our rich cohesive sensory experiences.

## Introduction

A withstanding cardinal question in neurophysiology is how neural circuits in the cerebral cortex compute and construct our perceptions of the world based on dynamically changing activity patterns of sensory neurons. One fundamental task faced by the visual system is the computation of global motion of an object from the collection of local motion of the objects constituents (Movshon et al., 1985; Newsome et al., 1990). Such a task is not trivial; when seen through an aperture, as imposed by the small receptive field of a retinal ganglion cell or a primary visual cortex (V1) neuron, only the motion component orthogonal to a contour can be inferred, while the parallel motion component remains ambiguous (Adelson and Movshon, 1982; Movshon et al., 1985; Carandini et al., 2005). Due to this “*aperture problem”*, one-dimensional local motion information from multiple contours needs to converge to unambiguously encode global two-dimensional object motion. To solve this challenging computational problem, it is generally assumed that visual circuits from retina to V1 first dissects direction of motion for components of the object, such as oriented contours (Yonehara et al., 2011, 2013; Cruz-Martín et al., 2014; Hillier et al., 2017) and neurons in extrastriate areas combine those component motions to form the global object motion representation (Adelson and Bergen, 1985; DeAngelis et al., 1993b; Albright and Stoner, 1995; Simoncelli and Heeger, 1998; Rust et al., 2006).

Global motion computations have been studied in a variety of animal species from flies (Saleem et al., 2012) to humans (Adelson and Movshon, 1982). In particular, non-human primates have been extensively used as a model (Movshon et al., 1985; Newsome and Paré, 1988; Britten et al., 1992; Tinsley et al., 2003; Smith et al., 2005; Majaj et al., 2007; Hedges et al., 2011; Solomon et al., 2011; Kumbhani et al., 2015; Chaplin et al., 2017), yielding seminal insights. Only now, are mice being investigated (Juavinett and Callaway, 2015; Muir et al., 2015; Palagina et al., 2017). The mouse offers several experimental advantages to primates and serves as an attractive model for elucidating the circuitry and single neuron computations underlying global motion processing, by granting access to the large genetic-, viral- and imaging toolboxes now available (Wickersham et al., 2007; Luo et al., 2008; Chen et al., 2013; Niell, 2015; Hawrylycz et al., 2016).

Here, we aim to provide a brief overview of the current experimental and theoretical literature on global motion processing based on works in primates and mice. Finally, we propose what experiments could propel the field forward and shed light on what circuit mechanisms are employed for this well-defined neural computation, by exploiting the mouse visual cortex as a model system.

## Local and Global Motion Is Encoded by Two Groups of Cortical Neurons

**Figure 1:**
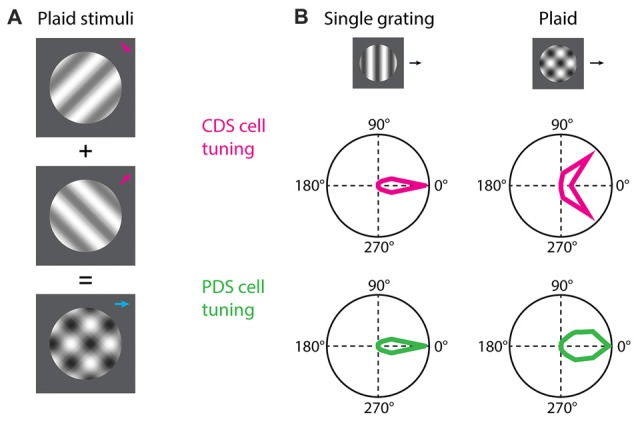
Component and pattern direction-selective (PDS) cells. **(A)** For the study of component direction-selective (CDS) and PDS cells the common visual stimulus employed is plaids consisting of two drifting gratings superimposed additively and offset by an angle. **(B)** Both CDS and PDS cells are tuned for the direction of motion of a single drifting grating. When a plaid stimulus is presented the predicted behavior of a PDS cell is that the neuron integrates the motion signals and responds to the plaid as it does to the individual grating but with broader tuning. The CDS cells responds to the individual grating components of the plaid as if they were presented alone. Polar plots in **(B)** are based on Smith et al. (2005).

For studying neural encoding of global motion, the stimulus commonly employed is the additive plaid pattern (Adelson and Movshon, 1982; Movshon et al., 1985; Tinsley et al., 2003; Smith et al., 2005; Rust et al., 2006; Solomon et al., 2011; Juavinett and Callaway, 2015; Figure 1A). This stimulus is composed of two oriented drifting gratings, offset by an angle, whose directions of motion are symmetric relative to the coherent pattern motion (Adelson and Movshon, 1982; Muir et al., 2015; Figure 1A). By harnessing the local and global constituents of the plaid, foundational experiments have demonstrated the existence of two groups of cortical neurons, based on their response properties to plaids (Movshon et al., 1985; Smith et al., 2005; Solomon et al., 2011).

Neurons encoding local motion are called component direction-selective (CDS) cells (Movshon et al., 1985; DeAngelis et al., 1993a; Smith et al., 2005; Solomon et al., 2011). These are sensitive to the direction of motion for the individual gratings of the plaid, and respond when any one of the gratings is moving in its preferred direction (Figure 1B). These cells are observed both in V1 and extrastriate areas. On the other hand, neurons encoding global motion are called pattern direction-selective (PDS) cells (Movshon et al., 1985; Smith et al., 2005; Solomon et al., 2011). These cells are observed in the extrastriate middle temporal (MT) area (but see also Tinsley et al., 2003) and show sensitivity to the direction of motion of the plaid, and respond when the coherent motion of the plaid is moving in the preferred direction (Figure 1B).

Based on electrophysiology experiments and computational approaches, the current theory in primates for object motion representation is described with a two-stage model (Simoncelli and Heeger, 1998; Rust et al., 2006). The first stage involves a summation field, in which presynaptic neurons of a PDS cell encode local motion of oriented elements. These presynaptic neurons are hypothesized as CDS cells in V1, supported by evidence that V1 neurons projecting into MT are CDS (Movshon and Newsome, 1996) and PDS cells do not reach their fully selective state until 75 ms after the responses of CDS cells have stabilized (Smith et al., 2005). The PDS cells should receive excitatory inputs from CDS cells with a wide range of preferred directions to account for a wide tuning profile of PDS cells. The second stage involves a normalization stage, which helps to encode direction of global motion in PDS cells independently of local visual features (Zeki, 1974; Movshon et al., 1985; Movshon and Newsome, 1996; Carandini and Heeger, 2011). Normalization could explain various observed suppressions, such as cross-orientation suppression, within and across the receptive field of PDS cells (Britten and Heuer, 1999). It remains to be determined whether normalization operates exclusively on V1 neurons or also on MT neurons. Whilst substantial evidence supports this model, causal and mechanistic data on the computation performed by PDS cells still remains lacking.

## Studying Local and Global Motion in Mouse Visual Cortex

**Figure 2:**
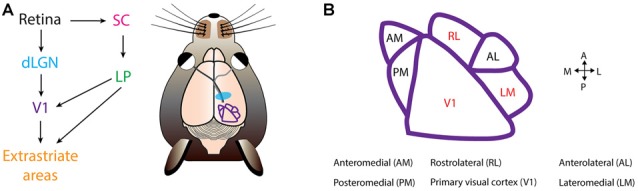
Mouse visual cortex organization. **(A)** The first step of visual processing occurs in the retina. The retina conveys visual information to the dorsal lateral geniculate nucleus (dLGN). From dLGN, information is transfered to primary visual cortex (V1), and from V1 information diverges, and is directed to extrastriate areas. In mice, another prominent pathway is from the retina to the superior colliculus (SC), and from SC further to the lateral posterior nucleus (LP), and LP finally projects into V1 and extrastriate areas. **(B)** Extrastriate areas receiving V1 projections include: anteromedial (AM), posteromedial (PM), rostrolateral (RL), anterolateral (AL) and lateromedial (LM). Currently, PDS cells have been located in V1, RL and LM (colored in red). Visual cortical map is based on Andermann et al. (2011).

At this time, three groups have probed the existence of CDS and PDS cells in mouse visual cortex (Juavinett and Callaway, 2015; Muir et al., 2015; Palagina et al., 2017). In the work by Juavinett and Callaway (2015), the proportion of PDS and CDS cells in layer 2/3 differed depending on the visual area. Mouse V1 is surrounded by extrastriate areas that receive V1 input (Wang and Burkhalter, 2007; Andermann et al., 2011; Marshel et al., 2011; Wang et al., 2012; Glickfeld et al., 2013a; Zhuang et al., 2017; Figure 2). The only areas that contained PDS cells were lateromedial (LM) and rostrolateral (RL; Figure 2). The fraction of PDS cells contained in LM and RL are 6% and 8%, respectively (Juavinett and Callaway, 2015). Both LM and RL are significantly interconnected with other visual areas (Wang et al., 2012), allowing them to combine inputs from many sources. It remains to be determined which mouse visual area is a homologous structure of MT, and it is possible this area lies outside the more commonly studied mouse extrastriate areas (Rosa, 1999). The other areas, including V1, contained no PDS cells, but only CDS cells (Juavinett and Callaway, 2015). However, others have suggested the existence of PDS cells in V1 (Muir et al., 2015; Palagina et al., 2017; Figure 2). The source of this discrepancy is unclear, but may partly originate from differences in plaid stimuli parameters. The work by Juavinett and Callaway (2015) presented additive plaids made from sinusoidal gratings, whereas the other works employed square-wave gratings for constructing the plaid (Muir et al., 2015; Palagina et al., 2017), yielding differences in spatial frequency content of the plaids. This could have potentially introduced differences in neuronal response properties. In congruence with this, human experiments have shown that the probability of a plaid percept is higher for square-wave gratings than for sinusoidal gratings (Burke et al., 1999). Another parameter is the temporal frequency of the plaid. PDS cells prefer drift rates of 2–16 Hz in non-human primates (Wang and Movshon, 2016). Juavinett and Callaway (2015) employed varying drift rates of 1, 1.5 or 2 Hz whereas Palagina et al. (2017) only used 2 Hz. The usage of low drift rates could have resulted in the underestimation of PDS responses in the work by Juavinett and Callaway (2015). In all instances, more experiments investigating the existence of PDS cells in V1 are needed by exploring the plaid parameter space. This current discrepancy also casts an important question to settle, as the existence of PDS cells at the stage of V1 may suggest that the two-stage model proposed in primates operates within V1 in mice, rather than exclusively across visual areas. Alternatively, PDS activity in V1 may be brought by recurrent projection of PDS cells in LM or RL, given significant interconnections between extrastriate areas and V1 (Wang et al., 2012; D’Souza et al., 2016). More detailed characterization of inter-areal functional connectivity would be crucial for answering this question, and could be achieved by recently developed wide-field two photon imaging methods (Stirman et al., 2016a) combined with high-speed recording of neuronal spikes with voltage sensors for understanding connection hierarchies (Gong et al., 2015).

Overall, evidence exist that mouse visual cortex represents and computes local and global motion, and therefore, is a valid model system for studying biophysical and circuit mechanisms of global motion computations in great detail. However, it may be plausible, and not all that surprising, if details in the strategy employed by mouse visual cortex for computing global motion deviates from that employed by humans and non-human primates. We speculate that primates and mice may have fundamental differences in the computational strategy and behavioral requirements of pattern motion computations. First of all, mice lack fovea in their retina unlike primates. Primate MT has a marked emphasis on the fovea; the central 15° of the visual field occupies over half of MT’s surface area (Van Essen et al., 1981), and signals from MT is important for the initiation of smooth pursuit, which is an eye movement for fixing a moving object on the fovea (Lisberger et al., 1987). Hence, pattern motion computation in mice would be less relevant for the smooth pursuit. Second, one of the most intriguing functional aspects of MT in primates is sensitivity to binocular disparity and depth perception. Since binocular areas are much smaller in mice compared to primates, pattern motion computations of mice may be specialized more for analyzing monocular motion, such as optic flow while running forward.

The functional organization of mouse and primate visual cortex differs on several levels (Huberman and Niell, 2011; Laramée and Boire, 2014). In rodent visual cortex, unlike primates, functionally selective cells are organized in a “*salt-and-pepper*” manner (Ohki et al., 2005; Bonin et al., 2011) and neurons with similar tuning are connected with each other (Ko et al., 2011; Li et al., 2012; Lee et al., 2016) in a layer-specific manner (Wertz et al., 2015) to form recurrent sub-networks. Differences upstream to visual cortex also exist. Given the high prevalence of direction-selective retinal ganglion cells (Vaney et al., 2012; Yonehara et al., 2013, 2016) and the finding of direction-selective dorsal lateral geniculate nucleus (dLGN) neurons and their feed-forward input to V1 (Cruz-Martín et al., 2014; Hillier et al., 2017), one might entertain the hypothesis that in mouse visual cortex, complex visual representations such as pattern motion develops at the stage of V1.

## Future Directions for Studying Local and Global Motion Processing in the Mouse

We propose five key questions to be addressed where mouse visual cortex would serve as an excellent model. Note however, recent advances have introduced the marmoset monkey as an attractive primate model due to its rapidly evolving molecular and genetic toolbox available (Sadakane et al., 2015a,b; Ding et al., 2017) and the organizational similarities to the visual system in humans. Furthermore, it might be advantageous to complement these experiments with studies in a rodent species in which homologs to MT have already been identified, such as the squirrel (Paolini and Sereno, 1998).

First question is: what is the tuning of individual excitatory synaptic inputs onto PDS cells when single gratings or plaids are shown? Models derived from non-human primate research (Simoncelli and Heeger, 1998; Rust et al., 2006) predict that PDS cells receive direct feed-forward excitatory barrages from CDS cells, and robust responses to plaids likely arises from convergence of CDS inputs tuned for different directions (Rust et al., 2006; Figure 1B). This question can be addressed by advanced methodologies such as dendritic spine calcium imaging (Jia et al., 2010; Chen et al., 2013; Wilson et al., 2016; Iacaruso et al., 2017). Dendritic spine calcium signals can serve as a proxy for the activity of individual excitatory synaptic inputs, and hence allows tuning characterization of incoming barrages.

Second question is: at which synaptic stages does normalization operate? Normalization is a fundamental neuronal computation that operates throughout the visual system and in many other sensory modalities (Carandini and Heeger, 2011). Normalization is an essential aspect of PDS cell behavior (Simoncelli and Heeger, 1998). This question could be addressed again by dendritic spine calcium imaging; one could test whether cross-orientation stimuli suppresses the activity of individual synaptic inputs to PDS cells or PDS somatic activity without suppressing synaptic inputs. In relation to these questions, it remains to be determined which brain areas provide synaptic inputs to PDS cells. In primates, MT receives inputs mainly from V1 and inferior pulvinar nucleus (Lyon et al., 2010), as well as short latency retinal inputs via the LGN (Warner et al., 2010). For this question, single-cell-initiated, activity sensor-functionalized monosynaptic tracing combined with network calcium imaging (Yonehara et al., 2013; Wertz et al., 2015) would be a powerful approach.

Third question is: what kind of dendritic mechanisms in PDS cells are involved in the integration of synaptic inputs? The response properties of PDS cells seem to be predicted from supra-linear summation of excitatory inputs (Muir et al., 2015). Recording of neuronal membrane potentials as well as excitatory and inhibitory synaptic currents by *in vivo* whole-cell patch-clamp recordings (Haider et al., 2016; Adesnik, 2017; Petersen, 2017) may provide hints for the answer.

Fourth question is: what is the role of brain state on PDS cell tuning? Recordings from CDS and PDS cells are often performed in anesthetized animals (Movshon et al., 1985; Tinsley et al., 2003; Smith et al., 2005; Solomon et al., 2011; Juavinett and Callaway, 2015; Palagina et al., 2017). However, it is now established that sensory experiences are shaped by the level of arousal, alertness and context (Albright and Stoner, 2002; Harris and Thiele, 2011; Keller et al., 2012; Lee and Dan, 2012; Lee et al., 2014; McGinley et al., 2015; Vinck et al., 2015). Motivating this question is recordings from MT (Pack et al., 2001), where the fraction of PDS cells were reduced in anesthetized animals compared to awake animals. These findings may suggest existence of top-down modulation of PDS cell tuning (Dent et al., 2010; Zhang et al., 2014). However, it should be noted that these findings have later been questioned in the field (Movshon et al., 2003). Here it was proposed that use of non-additive plaids could have affected the findings by Pack et al. (2001), leading to the discrepancy with previous work (Rodman and Albright, 1989; Stoner and Albright, 1992). Currently, there is no evidence that wakefulness changes the proportion of PDS cells in RL in mice (Juavinett and Callaway, 2015). Future experiments could address this question by performing population two photon calcium imaging in awake mice, for example, in a closed loop virtual reality, in which plaids motion coupled to locomotion are presented (Harvey et al., 2009; Keller et al., 2012; Roth et al., 2015).

Last question is: what is the role of PDS cells in perception and behavior? Previous work has implicated MT in psychophysical performance on object motion discrimination tasks (Newsome et al., 1990) and eye movement control (Newsome et al., 1985). However, whether PDS cells are the underlying biophysical substrate for the ability to discriminate object motion is unsettled (Tailby et al., 2010). Mice are capable of learning to discriminate between orientations or random-dot motion (Glickfeld et al., 2013b; Stirman et al., 2016b), allowing the possibility to track neural discriminability and tuning selectivity during learning of a visual task. We propose experiments where mice are trained to learn to discriminate plaid motion directions or global dot motion in more-or-less noisy conditions (Newsome and Paré, 1988), while two photon calcium imaging from PDS cells is simultaneously obtained, to directly compare neurometric and psychometric functions. Such experiments would provide correlational insights to the involvement of PDS cells in successful object motion discrimination and development of pattern motion selectivity over the learning period. Causality could be tested by single cell manipulation techniques such as two photon holographic optogenetics while performing the task (Packer et al., 2015; Carrillo-Reid et al., 2016; Dal Maschio et al., 2017).

Chemical lesion of MT caused deficits in smooth pursuit eye movements, important for following moving objects (Newsome et al., 1985). In concert with this, MT is known to project to several eye movement-related areas such as medial superior temporal cortex and pretectal nucleus of the optic tract (Mustari et al., 2009). In humans, perception of motion direction is well matched with the direction of fixation eye movements (Laubrock et al., 2008; Baker and Graf, 2010). Head or eye movements of mice could be used as a proxy for the output of cortical motion processing. Lastly, in a natural environment, global motion would be encountered frequently as an optic flow when a mouse is coursing through the environment. It may be important to bear the ethological point of view in mind when investigating particular aspects of visual function in the particular animal model employed (Huberman and Niell, 2011; Hillier et al., 2017).

## Conclusion

How PDS cells develop their distinct response properties to single gratings and patterned plaids is still an open question. Due to the rapidly evolving ability to interrogate neural circuits and single neuron computations using genetic and molecular techniques, PDS cells in mouse visual cortex are the perfect arena for delineating and solving how individual sensory features extracted by neuronal circuits in earlier brain regions are integrated to build our rich cohesive sensory experiences.

## Author Contributions

RR and KY drafted the manuscript, edited and revised the manuscript, and approved final version of the manuscript.

## Conflict of Interest Statement

The authors declare that the research was conducted in the absence of any commercial or financial relationships that could be construed as a potential conflict of interest.

## References

[B1] Adelson E. H. Bergen J. R. (1985). Spatiotemporal energy models for the perception of motion. J. Opt. Soc. Am. A 2, 284–299. 10.1364/josaa.2.000284 3973762

[B2] Adelson E. H. Movshon J. A. (1982). Phenomenal coherence of moving visual patterns. Nature 300, 523–525. 10.1038/300523a0 7144903

[B3] Adesnik H. (2017). Synaptic mechanisms of feature coding in the visual cortex of awake mice. Neuron 95, 1147.e4–1159.e4. 10.1016/j.neuron.2017.08.014 28858618 PMC5580349

[B4] Albright T. D. Stoner G. R. (1995). Visual motion perception. Proc. Natl. Acad. Sci. U S A 92, 2433–2440. 10.1073/pnas.92.7.2433 7708660 PMC42232

[B5] Albright T. D. Stoner G. R. (2002). Contextual influences on visual processing. Annu. Rev. Neurosci. 25, 339–379. 10.1146/annurev.neuro.25.112701.142900 12052913

[B6] Andermann M. L. Kerlin A. M. Roumis D. K. Glickfeld L. L. Reid R. C. (2011). Functional specialization of mouse higher visual cortical areas. Neuron 72, 1025–1039. 10.1016/j.neuron.2011.11.013 22196337 PMC3876958

[B7] Baker D. H. Graf E. W. (2010). Extrinsic factors in the perception of bistable motion stimuli. Vision Res. 50, 1257–1265. 10.1016/j.visres.2010.04.016 20433864

[B8] Bonin V. Histed M. H. Yurgenson S. Reid R. C. (2011). Local diversity and fine-scale organization of receptive fields in mouse visual cortex. J. Neurosci. 31, 18506–18521. 10.1523/JNEUROSCI.2974-11.2011 22171051 PMC3758577

[B9] Britten K. H. Heuer H. W. (1999). Spatial summation in the receptive fields of MT neurons. J. Neurosci. 19, 5074–5084. 10366640 10.1523/JNEUROSCI.19-12-05074.1999 PMC6782635

[B10] Britten K. H. Shadlen M. N. Newsome W. T. Movshon J. A. (1992). The analysis of visual motion: a comparison of neuronal and psychophysical performance. J. Neurosci. 12, 4745–4765. 1464765 10.1523/JNEUROSCI.12-12-04745.1992 PMC6575768

[B11] Burke D. Alais D. Wenderoth P. (1999). Determinants of fusion of dichoptically presented orthogonal gratings. Perception 28, 73–88. 10.1068/p2694 10627854

[B13] Carandini M. Demb J. B. Mante V. Tolhurst D. J. Dan Y. Olshausen B. A.. (2005). Do we know what the early visual system does? J. Neurosci. 25, 10577–10597. 10.1523/JNEUROSCI.3726-05.2005 16291931 PMC6725861

[B12] Carandini M. Heeger D. J. (2011). Normalization as a canonical neural computation. Nat. Rev. Neurosci. 13, 51–62. 10.1038/nrn3136 22108672 PMC3273486

[B14] Carrillo-Reid L. Yang W. Bando Y. Peterka D. S. Yuste R. (2016). Imprinting and recalling cortical ensembles. Science 353, 691–694. 10.1126/science.aaf7560 27516599 PMC5482530

[B15] Chaplin T. A. Allitt B. J. Hagan M. A. Price N. S. C. Rajan R. Rosa M. G. P.. (2017). Sensitivity of neurons in the middle temporal area of marmoset monkeys to random dot motion. J. Neurophysiol. 118, 1567–1580. 10.1152/jn.00065.2017 28637812 PMC5596136

[B16] Chen T.-W. Wardill T. J. Sun Y. Pulver S. R. Renninger S. L. Baohan A.. (2013). Ultrasensitive fluorescent proteins for imaging neuronal activity. Nature 499, 295–300. 10.1038/nature12354 23868258 PMC3777791

[B17] Cruz-Martín A. El-Danaf R. N. Osakada F. Sriram B. Dhande O. S. Nguyen P. L.. (2014). A dedicated circuit links direction-selective retinal ganglion cells to the primary visual cortex. Nature 507, 358–361. 10.1038/nature12989 24572358 PMC4143386

[B18] D’Souza R. D. Meier A. M. Bista P. Wang Q. Burkhalter A. (2016). Recruitment of inhibition and excitation across mouse visual cortex depends on the hierarchy of interconnecting areas. Elife 5:e19332. 10.7554/eLife.19332 27669144 PMC5074802

[B19] Dal Maschio M. Donovan J. C. Helmbrecht T. O. Baier H. (2017). Linking neurons to network function and behavior by two-photon holographic optogenetics and volumetric imaging. Neuron 94, 774.e5–789.e5. 10.1016/j.neuron.2017.04.034 28521132

[B20] DeAngelis G. C. Ohzawa I. Freeman R. D. (1993a). Spatiotemporal organization of simple-cell receptive fields in the cat’s striate cortex. I. General characteristics and postnatal development. J. Neurophysiol. 69, 1091–1117. 8492151 10.1152/jn.1993.69.4.1091

[B21] DeAngelis G. C. Ohzawa I. Freeman R. D. (1993b). Spatiotemporal organization of simple-cell receptive fields in the cat’s striate cortex. II. Linearity of temporal and spatial summation. J. Neurophysiol. 69, 1118–1135. 8492152 10.1152/jn.1993.69.4.1118

[B22] Dent K. Lestou V. Humphreys G. W. (2010). Deficits in visual search for conjunctions of motion and form after parietal damage but with spared hMT+/V5. Cogn. Neuropsychol. 27, 72–99. 10.1080/02643294.2010.497727 20665292

[B23] Ding R. Liao X. Li J. Zhang J. Wang M. Guang Y.. (2017). Targeted patching and dendritic Ca^2+^ imaging in nonhuman primate brain *in vivo*. Sci. Rep. 7:2873. 10.1038/s41598-017-03105-0 28588297 PMC5460116

[B24] Glickfeld L. L. Andermann M. L. Bonin V. Reid R. C. (2013a). Cortico-cortical projections in mouse visual cortex are functionally target specific. Nat. Neurosci. 16, 219–226. 10.1038/nn.3300 23292681 PMC3808876

[B25] Glickfeld L. L. Histed M. H. Maunsell J. H. R. (2013b). Mouse primary visual cortex is used to detect both orientation and contrast changes. J. Neurosci. 33, 19416–19422. 10.1523/JNEUROSCI.3560-13.2013 24336708 PMC3858618

[B26] Gong Y. Huang C. Li J. Z. Grewe B. F. Zhang Y. Eismann S.. (2015). High-speed recording of neural spikes in awake mice and flies with a fluorescent voltage sensor. Science 350, 1361–1366. 10.1126/science.aab0810 26586188 PMC4904846

[B27] Haider B. Schulz D. P. P. A. Häusser M. Carandini M. (2016). Millisecond coupling of local field potentials to synaptic currents in the awake visual cortex. Neuron 90, 35–42. 10.1016/j.neuron.2016.02.034 27021173 PMC4826437

[B28] Harris K. D. Thiele A. (2011). Cortical state and attention. Nat. Rev. Neurosci. 12, 509–523. 10.1038/nrn3084 21829219 PMC3324821

[B29] Harvey C. D. Collman F. Dombeck D. A. Tank D. W. (2009). Intracellular dynamics of hippocampal place cells during virtual navigation. Nature 461, 941–946. 10.1038/nature08499 19829374 PMC2771429

[B30] Hawrylycz M. Anastassiou C. Arkhipov A. Berg J. Buice M. Cain N.. (2016). Inferring cortical function in the mouse visual system through large-scale systems neuroscience. Proc. Natl. Acad. Sci. U S A 113, 7337–7344. 10.1073/pnas.1512901113 27382147 PMC4941493

[B31] Hedges J. H. Gartshteyn Y. Kohn A. Rust N. C. Shadlen M. N. Newsome W. T.. (2011). Dissociation of neuronal and psychophysical responses to local and global motion. Curr. Biol. 21, 2023–2028. 10.1016/j.cub.2011.10.049 22153156 PMC3241977

[B32] Hillier D. Fiscella M. Drinnenberg A. Trenholm S. Rompani S. B. Raics Z.. (2017). Causal evidence for retina-dependent and -independent visual motion computations in mouse cortex. Nat. Neurosci. 20, 960–968. 10.1038/nn.4566 28530661 PMC5490790

[B34] Huberman A. D. Niell C. M. (2011). What can mice tell us about how vision works? Trends Neurosci. 34, 464–473. 10.1016/j.tins.2011.07.002 21840069 PMC3371366

[B35] Iacaruso M. F. Gasler I. T. Hofer S. B. (2017). Synaptic organization of visual space in primary visual cortex. Nature 547, 449–452. 10.1038/nature23019 28700575 PMC5533220

[B36] Jia H. Rochefort N. L. Chen X. Konnerth A. (2010). Dendritic organization of sensory input to cortical neurons *in vivo*. Nature 464, 1307–1312. 10.1038/nature08947 20428163

[B37] Juavinett A. L. Callaway E. M. (2015). Pattern and component motion responses in mouse visual cortical areas. Curr. Biol. 25, 1759–1764. 10.1016/j.cub.2015.05.028 26073133 PMC4489988

[B38] Keller G. B. Bonhoeffer T. Hübener M. (2012). Sensorimotor mismatch signals in primary visual cortex of the behaving mouse. Neuron 74, 809–815. 10.1016/j.neuron.2012.03.040 22681686

[B39] Ko H. Hofer S. B. Pichler B. Buchanan K. A. Sjöström P. J. Mrsic-Flogel T. D. (2011). Functional specificity of local synaptic connections in neocortical networks. Nature 473, 87–91. 10.1038/nature09880 21478872 PMC3089591

[B40] Kumbhani R. D. El-Shamayleh Y. Movshon J. A. (2015). Temporal and spatial limits of pattern motion sensitivity in macaque MT neurons. J. Neurophysiol. 133, 1977–1988. 10.1152/jn.00597.2014 25540222 PMC4416600

[B41] Laramée M.-E. Boire D. (2014). Visual cortical areas of the mouse: comparison of parcellation and network structure with primates. Front. Neural Circuits 8:149. 10.3389/fncir.2014.00149 25620914 PMC4286719

[B42] Laubrock J. Engbert R. Kliegl R. (2008). Fixational eye movements predict the perceived direction of ambiguous apparent motion. J. Vis. 8:13. 10.1167/8.14.13 19146314

[B44] Lee S.-H. H. Dan Y. (2012). Neuromodulation of brain states. Neuron 76, 109–222. 10.1016/j.neuron.2012.09.012 23040816 PMC3579548

[B45] Lee W. A. Bonin V. Reed M. Graham B. J. Hood G. Glattfelder K.. (2016). Anatomy and function of an excitatory network in the visual cortex. Nature 532, 370–374. 10.1038/nature17192 27018655 PMC4844839

[B43] Lee A. M. Hoy J. L. Bonci A. Wilbrecht L. Stryker M. P. Niell C. M. (2014). Identification of a brainstem circuit regulating visual cortical state in parallel with locomotion. Neuron 83, 455–466. 10.1016/j.neuron.2014.06.031 25033185 PMC4151326

[B46] Li Y. Lu H. Cheng P. L. Ge S. Xu H. Shi S. H.. (2012). Clonally related visual cortical neurons show similar stimulus feature selectivity. Nature 486, 118–121. 10.1038/nature11110 22678292 PMC3375857

[B47] Lisberger S. G. Morris E. J. Tychsen L. (1987). Visual motion processing and sensory-motor integration for smooth pursuit eye movements. Annu. Rev. Neurosci. 10, 97–129. 10.1146/annurev.neuro.10.1.97 3551767

[B48] Luo L. Callaway E. M. Svoboda K. (2008). Genetic dissection of neural circuits. Neuron 57, 634–660. 10.1016/j.neuron.2008.01.002 18341986 PMC2628815

[B110] Lyon D. C. Nassi J. J. Callaway E. M. (2010). A disynaptic relay from superior colliculus to dorsal stream visual cortex in macaque monkey. Neuron 65, 270–279. 10.1016/j.neuron.2010.01.003 20152132 PMC2832737

[B49] Majaj N. J. Carandini M. Movshon J. A. (2007). Motion integration by neurons in macaque MT is local, not global. J. Neurosci. 27, 366–370. 10.1523/JNEUROSCI.3183-06.2007 17215397 PMC3039841

[B50] Marshel J. H. Garrett M. E. Nauhaus I. Callaway E. M. (2011). Functional specialization of seven mouse visual cortical areas. Neuron 72, 1040–1054. 10.1016/j.neuron.2011.12.004 22196338 PMC3248795

[B51] McGinley M. J. David S. V. McCormick D. A. (2015). Cortical membrane potential signature of optimal states for sensory signal detection. Neuron 87, 179–192. 10.1016/j.neuron.2015.05.038 26074005 PMC4631312

[B52] Movshon J. A. Adelson E. H. Gizzi M. S. Newsome W. T. (1985). The analysis of moving visual patterns. Pattern Recognit. Mech. 54, 117–151. 10.1007/978-3-662-09224-8_7

[B53] Movshon J. A. Albright T. D. Stoner G. R. Majaj N. J. (2003). Cortical responses to visual motion in alert and anesthetized monkeys. Nat. Neurosci. 6:3. 10.1038/nn0103-3b 12494238

[B54] Movshon J. A. Newsome W. T. (1996). Visual response properties of striate cortical neurons projecting to area MT in macaque monkeys. J. Neurosci. 16, 7733–7741. 8922429 10.1523/JNEUROSCI.16-23-07733.1996 PMC6579106

[B55] Muir D. R. Roth M. M. Helmchen F. Kampa B. M. (2015). Model-based analysis of pattern motion processing in mouse primary visual cortex. Front. Neural Circuits 9:38. 10.3389/fncir.2015.00038 26300738 PMC4525018

[B56] Mustari M. J. Ono S. Das V. E. (2009). Signal processing and distribution in cortical-brainstem pathways for smooth pursuit eye movements. in. Ann. N Y Acad. Sci. 1164, 147–154. 10.1111/j.1749-6632.2009.03859.x 19645893 PMC3057571

[B58] Newsome W. T. Britten K. H. Salzman C. D. Movshon J. A. (1990). Neuronal mechanisms of motion perception. Cold Spring Harb. Symp. Quant. Biol. 55, 697–705. 10.1101/sqb.1990.055.01.065 2132847

[B57] Newsome W. T. Paré E. B. (1988). A selective impairment of motion perception following lesions of the middle temporal visual area (MT). J. Neurosci. 8, 2201–2211. 3385495 10.1523/JNEUROSCI.08-06-02201.1988 PMC6569328

[B59] Newsome W. Wurtz R. Dürsteler M. Mikami A. (1985). Deficits in visual motion processing following ibotenic acid lesions of the middle temporal visual area of the macaque monkey. J. Neurosci. 5, 825–840. 3973698 10.1523/JNEUROSCI.05-03-00825.1985 PMC6565029

[B60] Niell C. M. (2015). Cell types, circuits, and receptive fields in the mouse visual cortex. Annu. Rev. Neurosci. 38, 413–431. 10.1146/annurev-neuro-071714-033807 25938727

[B61] Ohki K. Chung S. Ch’ng Y. H. Kara P. Reid R. C. (2005). Functional imaging with cellular resolution reveals precise micro-architecture in visual cortex. Nature 433, 597–603. 10.1038/nature03274 15660108

[B62] Pack C. C. Berezovskii V. K. Born R. T. (2001). Dynamic properties of neurons in cortical area MT in alert and anaesthetized macaque monkeys. Nature 414, 905–908. 10.1038/414905a 11780062

[B63] Packer A. M. Russell L. E. Dalgleish H. W. P. Häusser M. (2015). Simultaneous all-optical manipulation and recording of neural circuit activity with cellular resolution *in vivo*. Nat. Methods 12, 140–146. 10.1038/nmeth.3217 25532138 PMC4933203

[B64] Palagina G. Meyer J. F. Smirnakis S. M. (2017). Complex visual motion representation in mouse area V1. J. Neurosci. 37, 164–183. 10.1523/JNEUROSCI.0997-16.2017 28053039 PMC5214628

[B65] Paolini M. Sereno M. I. (1998). Direction selectivity in the middle lateral and lateral (ML and L) visual areas in the California ground squirrel. Cereb. Cortex 8, 362–371. 10.1093/cercor/8.4.362 9651131

[B66] Petersen C. C. H. (2017). Whole-cell recording of neuronal membrane potential during behavior. Neuron 95, 1266–1281. 10.1016/j.neuron.2017.06.049 28910617

[B67] Rodman H. R. Albright T. D. (1989). Single-unit analysis of pattern-motion selective properties in the middle temporal visual area (MT). Exp. Brain Res. 75, 53–64. 10.1007/bf00248530 2707356

[B68] Rosa M. G. P. (1999). Topographic organisation of extrastriate areas in the flying fox: implications for the evolution of mammalian visual cortex. J. Comp. Neurol. 411, 503–523. 10.1002/(sici)1096-9861(19990830)411:3<503::aid-cne12>3.0.co;2-6 10413783

[B69] Roth M. M. Dahmen J. C. Muir D. R. Imhof F. Martini F. J. Hofer S. B. (2015). Thalamic nuclei convey diverse contextual information to layer 1 of visual cortex. Nat. Neurosci. 19, 299–307. 10.1038/nn.4197 26691828 PMC5480596

[B70] Rust N. C. Mante V. Simoncelli E. P. Movshon J. A. (2006). How MT cells analyze the motion of visual patterns. Nat. Neurosci. 9, 1421–1431. 10.1038/nn1786 17041595

[B71] Sadakane O. Masamizu Y. Watakabe A. Terada S. I. Ohtsuka M. Takaji M.. (2015a). Long-term two-photon calcium imaging of neuronal populations with subcellular resolution in adult non-human primates. Cell Rep. 13, 1989–1999. 10.1016/j.celrep.2015.10.050 26655910

[B72] Sadakane O. Watakabe A. Ohtsuka M. Takaji M. Sasaki T. Kasai M.. (2015b). *In vivo* two-photon imaging of dendritic spines in marmoset neocortex (1,2,3). eNeuro 2:ENEURO.0019-15.2015. 10.1523/eneuro.0019-15.2015 26465000 PMC4596018

[B73] Saleem A. B. Longden K. D. Schwyn D. A. Krapp H. G. Schultz S. R. (2012). Bimodal optomotor response to plaids in blowflies: mechanisms of component selectivity and evidence for pattern selectivity. J. Neurosci. 32, 1634–1642. 10.1523/JNEUROSCI.4940-11.2012 22302805 PMC6703340

[B74] Simoncelli E. P. Heeger D. J. (1998). A model of neuronal responses in visual area MT. Vision Res. 38, 743–761. 10.1016/s0042-6989(97)00183-1 9604103

[B75] Smith M. A. Majaj N. J. Movshon J. A. (2005). Dynamics of motion signaling by neurons in macaque area MT. Nat. Neurosci. 8, 220–228. 10.1038/nn1382 15657600

[B76] Solomon S. S. Tailby C. Gharaei S. Camp A. J. Bourne J. A. Solomon S. G. (2011). Visual motion integration by neurons in the middle temporal area of a New World monkey, the marmoset. J. Physiol. 589, 5741–5758. 10.1113/jphysiol.2011.213520 21946851 PMC3249047

[B77] Stirman J. N. Smith I. T. Kudenov M. W. Smith S. L. (2016a). Wide field-of-view, multi-region, two-photon imaging of neuronal activity in the mammalian brain. Nat. Biotechnol. 34, 857–862. 10.1038/nbt.3594 27347754 PMC4980167

[B78] Stirman J. N. Townsend L. B. Smith S. L. (2016b). A touchscreen based global motion perception task for mice. Vision Res. 127, 74–83. 10.1016/j.visres.2016.07.006 27497283 PMC5035629

[B79] Stoner G. R. Albright T. D. (1992). Neural correlates of perceptual motion coherence. Nature 358, 412–414. 10.1038/358412a0 1641024

[B80] Tailby C. Majaj N. J. Movshon J. A. (2010). Binocular integration of pattern motion signals by MT neurons and by human observers. J. Neurosci. 30, 7344–7349. 10.1523/JNEUROSCI.4552-09.2010 20505101 PMC2893719

[B81] Tinsley C. J. Webb B. S. Barraclough N. E. Vincent C. J. Parker A. Derrington A. M. (2003). The nature of V1 neural responses to 2D moving patterns depends on receptive-field structure in the marmoset monkey. J. Neurophysiol. 90, 930–937. 10.1152/jn.00708.2002 12711710

[B82] Van Essen D. C. Maunsell J. H. Bixby J. L. (1981). The middle temporal visual area in the macaque: myeloarchitecture, connections, functional properties and topographic organization. J. Comp. Neurol. 199, 293–326. 10.1002/cne.901990302 7263951

[B83] Vaney D. I. Sivyer B. Taylor W. R. (2012). Direction selectivity in the retina: symmetry and asymmetry in structure and function. Nat. Rev. Neurosci. 13, 194–208. 10.1038/nrn3165 22314444

[B84] Vinck M. Batista-Brito R. Knoblich U. Cardin J. A. (2015). Arousal and locomotion make distinct contributions to cortical activity patterns and visual encoding. Neuron 86, 740–754. 10.1016/j.neuron.2015.03.028 25892300 PMC4425590

[B86] Wang Q. Burkhalter A. (2007). Area map of mouse visual cortex. J. Comp. Neurol. 502, 339–357. 10.1002/cne.21286 17366604

[B85] Wang H. X. Movshon J. A. (2016). Properties of pattern and component direction-selective cells in area MT of the macaque. J. Neurophysiol. 115, 2705–2720. 10.1152/jn.00639.2014 26561603 PMC4922598

[B87] Wang Q. Sporns O. Burkhalter A. (2012). Network analysis of corticocortical connections reveals ventral and dorsal processing streams in mouse visual cortex. J. Neurosci. 32, 4386–4399. 10.1523/jneurosci.6063-11.2012 22457489 PMC3328193

[B88] Warner C. E. Goldshmit Y. Bourne J. A. (2010). Retinal afferents synapse with relay cells targeting the middle temporal area in the pulvinar and lateral geniculate nuclei. Front. Neuroanat. 4:8. 10.3389/neuro.05.008.2010 20179789 PMC2826187

[B89] Wertz A. Trenholm S. Yonehara K. Hillier D. Raics Z. Leinweber M.. (2015). Single-cell-initiated monosynapic tracing reveals layer-specific cortical network modules. Science 349, 70–74. 10.1126/science.aab1687 26138975

[B90] Wickersham I. R. Lyon D. C. Barnard R. J. O. Mori T. Finke S. Conzelmann K.-K.. (2007). Monosynaptic restriction of transsynaptic tracing from single, genetically targeted neurons. Neuron 53, 639–647. 10.1016/j.neuron.2007.01.033 17329205 PMC2629495

[B91] Wilson D. E. Whitney D. E. Scholl B. Fitzpatrick D. (2016). Orientation selectivity and the functional clustering of synaptic inputs in primary visual cortex. Nat. Neurosci. 19, 1003–1009. 10.1038/nn.4323 27294510 PMC5240628

[B92] Yonehara K. Balint K. Noda M. Nagel G. Bamberg E. Roska B. (2011). Spatially asymmetric reorganization of inhibition establishes a motion-sensitive circuit. Nature 469, 407–410. 10.1038/nature09711 21170022

[B93] Yonehara K. Farrow K. Ghanem A. Hillier D. Balint K. Teixeira M.. (2013). The first stage of cardinal direction selectivity is localized to the dendrites of retinal ganglion cells. Neuron 79, 1078–1085. 10.1016/j.neuron.2013.08.005 23973208

[B94] Yonehara K. Fiscella M. Drinnenberg A. Esposti F. Trenholm S. Krol J.. (2016). Congenital nystagmus gene FRMD7 is necessary for establishing a neuronal circuit asymmetry for direction selectivity. Neuron 89, 177–193. 10.1016/j.neuron.2015.11.032 26711119 PMC4712192

[B95] Zeki S. M. (1974). Functional organization of a visual area in the posterior bank of the superior temporal sulcus of the rhesus monkey. J. Physiol. 236, 549–573. 10.1113/jphysiol.1974.sp010452 4207129 PMC1350849

[B96] Zhang S. Xu M. Kamigaki T. Hoang Do J. P. Chang W.-C. Jenvay S.. (2014). Long-range and local circuits for top-down modulation of visual cortex processing. Science 345, 660–665. 10.1126/science.1254126 25104383 PMC5776147

[B97] Zhuang J. Ng L. Williams D. Valley M. Li Y. Garrett M.. (2017). An extended retinotopic map of mouse cortex. Elife 6:e18372. 10.7554/elife.18372 28059700 PMC5218535

